# Composting Performance of l-Poly(lactic acid), d-Poly(lactic acid), Their Blends, and Stereocomplex PLA Films

**DOI:** 10.1021/acsomega.5c00985

**Published:** 2025-05-01

**Authors:** James F. Macnamara, Anibal Bher, Rafael Auras

**Affiliations:** School of Packaging, Michigan State University, East Lansing, Michigan 48824-1223, United States

## Abstract

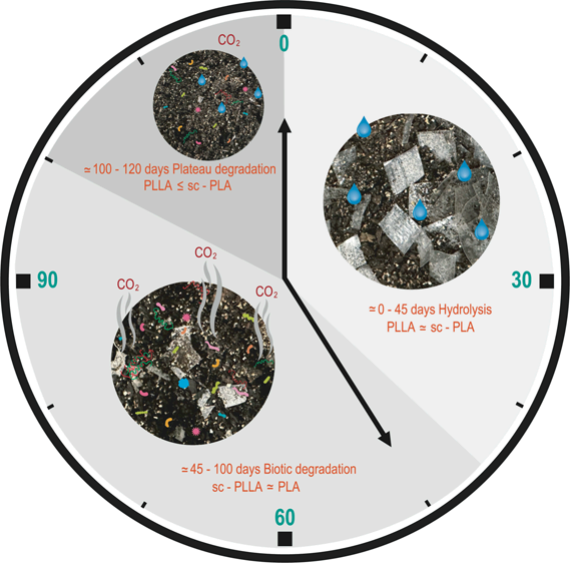

Single-use plastics are a major contributor to the generation of plastic waste. Biodegradable polymers provide some hope of curbing this emerging waste issue, with polylactic acid being a promising example. This study examined the biodegradation of l-poly(lactic acid)—PLLA, d-poly(lactic acid)—PDLA, various blends of PLLA and PDLA, as well as a sample of sc-PLLA/PDLA-50–50-A annealed for 30 min to induce crystallization. A simulated study in a lab-scale direct measurement respirometer compared the abiotic and biotic degradations of the various films in compost. The films’ crystallinity increased at the beginning of degradation before plateauing. The molecular weight (*M*_W_) decreased first due to hydrolysis from about days 30 to 60, depending on the film, and then due to biodegradation when the microorganisms were able to assimilate the oligomers after it was broken down sufficiently by hydrolysis. By 120 days, the percent biodegradation of the annealed sc-PLLA/PDLA-50–50-A was greatest, at 97%, followed closely by the PLLA/PDLA 50–50 blend at 86%, while PDLA biodegraded the least, at only 40%. Scanning electron microscopy micrographs obtained for all films from day 0 up to day 60 clearly show the erosion of the films over the experiment’s progression. These findings showcase the potential of stereocomplex PLA as a biodegradable plastic alternative and support the pursuit of complementing or replacing traditional petrochemical-based plastic options.

## 1 Introduction

Sustainable polymers are materials derived from renewable, recycled, and waste carbon resources that can be recycled, biodegraded, or composted at the end of life.^1^ Biodegradable polymers can be a subset of sustainable polymers that break down after their intended purpose by bacterial decomposition, resulting in natural byproducts, such as gases, water, biomass, and inorganic salts.^2^ Producing biodegradable, sustainable polymers requires a delicate balance between delivering functional performance properties and minimizing environmental impact at the end of life.^3^ For instance, there is a growing demand for materials that offer superior water barrier properties and enhanced thermal resistance. However, to promote both abiotic and biotic degradation at the end of their life cycle, these polymers must also be engineered for water susceptibility and increased chain mobility, enhancing their degradation potential.^4^ Thus, developing these sustainable polymers is a strategic endeavor that optimizes performance while ensuring effective recovery and environmental stewardship at the end of their lifecycle.

Biodegradation involves microorganisms decomposing organic materials into water, biomass, and CO_2._^2^ As global efforts to mitigate the environmental impact of single-use plastics intensify, biodegradable materials are gaining prominence across various sectors, including packaging and disposable serviceware. Recognizing the potential to reduce the environmental impact of single-use plastic products, governments worldwide are prioritizing the expansion of the biodegradable plastics market. A report by Allied Market Research projects a significant growth trajectory for the global bioplastics and biodegradable market, forecasting an increase from USD 1.6 billion in 2019 to USD 4.2 billion by 2026.^5^

Compostable plastics are a subset of biodegradable polymers, which can be derived from fossil or renewable sources.^4^ To be labeled as compostable, plastic must biodegrade under specific conditions within a particular time frame without leaving any toxic residue or chemicals.^6^ Various standards specify the criteria upon which a material must conform to be claimed to be industrial compostable. The primary standards are ASTM D6400-21^7^ and CEN EN 13432:2000.^8^ These standards have similar basic requirements: (a) disintegration during composting; (b) biodegradation in a set time frame compared with a readily biodegradable control such as cellulose; and (c) no adverse effects on the ability of the compost to support plant germination and growth.^9^

The composting environment presents an ideal solution for the disposal of materials unsuitable for recycling due to contamination that could disrupt the recycling process. This is especially relevant for food packaging items that should be sent elsewhere than landfills.^10^ These materials often include various types of single-use packaging and serviceware typically contaminated with food or viscous beverages, making composting a practical and environmentally responsible end-of-life option.

Poly(lactic acid) (PLA) is a biodegradable polymer derived from renewable resources, which decomposes under industrial composting conditions.^11^ The most commercially prevalent form of PLA, primarily consisting of a majority of l-PLA (PLLA), represents a promising alternative to fossil-based plastics, aiming to reduce the volume of nonbiodegradable packaging waste ending up in landfills.^12^ However, despite its environmental benefits, PLLA faces challenges such as a lower moisture barrier,^13^ low heat deflection temperature,^14^ and inferior hydrolytic and thermal stability^15^ compared to traditional fossil-based plastics.

PLA undergoes multiple phases during aerobic biodegradation, transitioning from an abiotic-dominant stage to a biotic-dominant stage. Initially, the abiotic phase is characterized by a lag period, in which the polymer resists mineralization. Following this lag phase, mineralization occurs predominantly through the biotic processes. This biodegradation process unfolds in several stages: (1) water diffuses into the amorphous regions of the polymer; (2) ester bond scission occurs in the amorphous chains, reducing *M*_W_ and generating oligomers and monomers; and (3) partial hydrolysis of the boundary of the crystalline phase.^16^ Once the average *M*_W_ drops below 10 kDa, biotic degradation commences, allowing microorganisms to break down the polymer.^17^ Up to that point, the size of the polymer chains is too large for the microorganisms to use as a food source. Before this point, the polymer chains were too large to be used for microbial consumption. Once microbial action is complete—marking the dominant biotic stage—the process enters a plateau phase, signaling the completion of biodegradation.^17^

Combining PLLA with the other enantiomer of PLA, d-PLA (PDLA), under specific processing conditions, can produce blends and stereocomplex PLA (sc-PLA), which has improved properties over homocomplex PLA (hc-PLA), namely, enhanced moisture vapor transmission rate (MVTR),^18^ increased heat resistance,^19,20^ and hydrolytic stability^21^; these are desired properties for single-use packaging and food/beverage contact applications. Much of the research on sc-PLA films has utilized solvent casting, which is impractical for commercial-scale production. Additionally, it has been reported that the annealing process of sc-PLA films can enhance the moisture barrier properties of these films by promoting crystallization.^20^

The properties of PDLA films have yet to be studied in most areas, including hydrolysis or biodegradation. Mbarki et al. compared the biodegradation of PDLA and PDLA/cellulose microfibers with a specific *Pseudomonas* strain at mesophilic temperatures. They determined that crystallinity was the principal factor influencing the biodegradation process. During the process, the PDLA/cellulose microfibers swelled less than PDLA by contact with water, hence having a slower breakdown rate.^22^ However, the study compared only PDLA to PDLA/cellulose microfibers. Tomita et al. reported on the degradation of PDLA by *Bacillus stearothermophilus* strain *#73.*(23) In their biodegradation study on PDLA with and without the strain, the sample with strain #73 proved biodegradable. Similar changes were seen in the sample without strain but were inferior to those seen in the run with the strain. The researchers did not include PLLA or any other material for comparison.

Our work uniquely addresses the interplay between polymers’ enhanced properties during their useful life and their subsequent end-of-life performance. We evaluate the biodegradation performance of previously developed PLLA/PDLA blends and PDLA produced via cast extrusion, along with one blend that has been annealed to induce crystallization and form sc-PLA.^20^ This research methodically analyzes the degradation process’s impact on *M*_W_, crystallinity, and surface deterioration. We examine multiple PLLA/PDLA blends, including an annealed version, over 120 days to assess the abiotic and biotic degradation process. We also include controls of PLLA and cellulose—both well-documented materials—to provide a comparative baseline. Notably, our work also introduces the biodegradation of PDLA films tested at thermophilic temperatures, a comparison not previously explored. This insight will guide the use of these polymers in contaminated single-use packaging, providing a viable biodegradable alternative to help reduce plastic waste and develop sustainable polymers.

## 2 Experimental Section

### 2.1 Materials

PLLA and PDLA resins were supplied by TotalEnergies Corbion (Gorinchem, The Netherlands). The specific grades used were Luminy L130 (≥99%(l-isomer)) and Luminy D120 (≥99%(d-isomer)). The resins were processed as received. Tetrahydrofuran (THF), used as the mobile solvent to dissolve the materials to determine the *M*_W_, was procured from Pharmco by Greenfield Global (Winnebago, MN, USA). Cellulose powder was procured from Sigma-Aldrich (St Louis, MO, USA). Mature compost was obtained from the Michigan State University (MSU) composting facility (East Lansing, MI, USA).

### 2.2 Film Processing

All of the films were produced on a pilot-scale cast film microextruder (Randcastle Extrusion Systems, Cedar Grove, NJ,USA). PLLA and PDLA films were extruded separately from the two dried resins. Then, the two resins were blended by weight in ratios of 70/30, 50/50, and 30/70 PLLA/PDLA before being introduced to the extruder. The processing conditions are detailed elsewhere.^20^

### 2.3 Thermal Annealing

The 50/50 PLLA/PDLA sample was annealed at 160 °C for 30 min to induce crystallization in a PHI hydraulic press model no. QL438-C (City of Industry, CA, USA). The size of the samples was approximately 25.4 × 16.5 cm^2^. The samples were placed between 25.4 × 25.4 cm^2^ plates lined with nonstick aluminum foil. After being annealed, the samples were cooled at ambient temperature and stored in a freezer at −20 °C before being prepared for the biodegradation experiment.

### 2.4 CHN Analysis

The samples’ carbon, hydrogen, and nitrogen amounts were analyzed using a CHNS/O Elemental Analyzer, 2400 Series II (PerkinElmer, Waltham, MA, USA). About 2 mg of each film sample was weighed in a small Sn capsule and analyzed. Triplicate measurements of each sample were taken. The electronic Supporting Information (ESI) Table S1 shows the carbon content values of the films used in the study.

### 2.5 Sample Preparation for Biodegradation

Some frozen film samples were ground into powder using a Single Speed Mini Cutting Mill (model E3300.00; Eberbach Corporation, Van Buren Township, MI, USA). A 20 mesh screen was used in the mill to allow only pieces with lower dimensions to pass through it. The samples for differential scanning calorimetry (DSC) and size exclusion chromatography (SEC) testing were cut into 1 × 1 cm^2^ squares since the powder could not be easily separated from the compost during biodegradation. At least 24 g of each powder and 8 g of each square per variable were prepared to test three bioreactors for biodegradation and one for sampling. The samples were then placed back into the freezer until the start of the biodegradation test.

### 2.6 Biodegradation Test in Compost

The biodegradation of PLLA, PDLA, several blends of PLLA/PDLA, and one sc-PLA (50/50) film was evaluated under aerobic conditions using a direct measurement respirometric (DMR) system at the MSU School of Packaging. The system has a nondispersive infrared gas analyzer (Li-Cor, Lincoln, NE, USA), which measures the CO_2_ that evolved throughout the experiment. The chamber’s temperature and relative humidity (RH) were maintained at 58 ± 2 °C and 50 ± 5% RH, respectively. The airflow rate was regulated at 40 ± 2 cm^3^/min. More information on the DMR system is detailed elsewhere.^24^

Manure compost was procured from the MSU composting facility and sifted through a 10 mm screen to remove large chunks of debris. The laboratory analysis of the compost is included in Table S2. The compost was adjusted to 50 ± 5% RH using deionized water. The screenings were then conditioned to 58 ± 2 °C. Each bioreactor was filled with 400 g of compost, and then, 8 g of each test sample variable was introduced to each bioreactor individually for testing in triplicate. A blank (only compost) and the positive control (cellulose) were included in the test.

During the test, deionized water was injected into the bioreactors weekly to maintain the moisture content at the optimal level. Air without CO_2_ was introduced to each bioreactor, and the amount of liberated CO_2_ was measured over a finite period. The system was purged after each measurement to eliminate any CO_2_ left over from the previous measurement and to maintain a clean baseline. The percentage of biodegradation, which is the amount of carbon transformed to CO_2_, was calculated from eq 1(24):
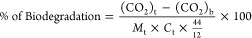
1where (CO_2_)_t_ is the average total CO_2_ evolved from the bioreactor containing the sample; (CO_2_)_b_ is the average total CO_2_ evolved from the blank; *M*_t_ is the total mass of the sample in the bioreactor; *C*_t_ is the total carbon content of the sample as measured by CHN analysis; 44 is the *M*_W_ of CO_2_, and 12 is the atomic weight of carbon.

### 2.7 Differential Scanning Calorimetry

Samples of all of the films from the biodegradation test at 0, 7, 14, 21, 28, and 45 days were tested using a Q100 DSC (TA Instruments, New Castle, DE, USA). PLLA and PDLA were also tested at 60 days. The calorimeter has a cooling system using a 70 mL/min nitrogen flow. Samples weighing 5–10 mg were packed and tested in standard aluminum pans and lids. The thermograms were collected from 20 to 260 °C at 10 °C/min for two cycles. The sample was held isothermally for 1 min at 260 °C between cycles. One or more replicates of each sample were tested on each film in the study at each period. The analysis was run on Universal Analysis 2000 software version 4.5A (TA Instruments) to collect the DSC thermograms.

### 2.8 Wide-Angle X-ray Diffraction

An AXS D8 Advance X-ray diffractometer (Bruker Co., Billerica, MA, USA) with a global mirror filter Cu Kα radiation source at 40 kV and 100 mA was used to collect wide-angle X-ray diffraction (WAXD) patterns. The data were collected between a 2θ range of 10° and 40° at a rate of 0.24° min^–1^ and an increment of 0.01°. DIFFRAC.MEASUREMENT CENTER version 7.5.0 software (Bruker Co.) was used to collect the data. DIFFRAC.EVA version 5.1.0.5 (Bruker Co., ) was used to evaluate the data generated from the DIFFRAC.MEASUREMENT CENTER software used for the WAXD patterns. The relative crystallinity was calculated from the ratio of the area under the crystalline peak to the total area of the crystalline and amorphous peaks. Fityk 1.3.1 software^25^ was used for background subtraction and diffractogram deconvolution using a Gaussian function. At each sample time, at least one film sample was tested.

### 2.9 Size Exclusion Chromatography

The number-average molecular weight (*M*_n_), weight-average molecular weight (*M*_w_), and molecular weight distribution (MWD) of the PLLA and PDLA samples at 0, 7, 14, 21, 28, 45, and 60 days were measured using an SEC system (Waters, New Castle, DE, USA). The SEC system has an autosampler, a refractive index detector, and an isocratic pump with a series of Styragel columns (Styragel HR-4, HR-3, and HR-2). Approximately 20 mg of each sample was dispersed in 10 mL of THF and stored overnight to dissolve. After the samples were left to sit overnight, they were placed into an oven at 80 °C to allow them to dissolve thoroughly. The samples were filtered, transferred to a 2 mL glass vial, and capped. The test was run at a temperature of 35 °C with a flow rate of 1 mL/min for 50 min for each sample. To determine sample *M*_n_*, M*_w_*,* and MWD, the Mark–Houwink constants used for PLA were *K* = 0.000174 dL/g and α = 0.736.^26^ Waters Breeze2 software was used to analyze the data. The experimental data were fitted using a first-order equation of *M*_n_/*M*_n0_ = e^(−*kt*)^, where *M*_n0_ is the *M*_n_ at day 0, *k* is the rate constant in 1/*d*, and *t* is the time in days.^27,28^ At least three replicates of each sample were measured. We could not test the PLLA/PDLA-blended samples since they do not dissolve in THF, and our SEC system exclusively runs THF.

### 2.10 Scanning Electron Microscopy

Surface scans of various films at day 0 and retrieved from biodegradation were captured using a scanning electron microscope (model JSM 6610LV; Jeol USA Inc., Peabody, MA, USA) to track the samples’ surface deterioration throughout the study. The scanning electron microscopy (SEM) operating conditions were an accelerated voltage of 12 kV, a spot size of 30, and a vacuum pressure of 1.33 × 10^–5^ Pa, with magnifications of 330 and 5000×, depending on the sample. The samples were sputtered with gold using a current of 20 mA for 3 min.

### 2.11 Data Analysis

The data and graphs were compiled and analyzed using Microsoft Excel (Microsoft, Redmond, WA, USA) and MATLAB 2024a (Mathworks, Natick, MA, USA).

## 3 Results and Discussion

The film samples for biodegradation were prepared, placed into the bioreactors, and mixed with compost before being put into the DMR. The test was conducted for 120 days, sampling at 7, 14, 21, 28, 45, and 60 days. After day 60, it was no longer possible to collect film samples; therefore, only CO_2_ measurements were collected. CO_2_ measurements were continually collected throughout the test to calculate the % biodegradation. Photos of the bioreactors containing the various films at each sampling point are shown in Figure S1. Results obtained for crystallinity, *M*_w_ evolution, and surface deterioration for samples at day 0 and as retrieved during biodegradation are presented and discussed below.

### 3.1 Crystallinity

We first tried to use DSC to determine the crystallinity of the samples. However, this analysis had issues due to the overlapping melting and crystalline peaks of hc-PLA and sc-PLA in the blended samples in the same region around 175 °C. The results are included in Figures S2 and S3. Zhang et al. studied the melting behavior of sc-crystals by DSC and time-resolved in situ WAXD produced by equimolar PLLA/PDLA blends prepared by a freeze-drying method from their solutions in 1,4-dioxane at different concentrations between 10,000 and 0.005 wt %.^29^ They demonstrated that the crystallization of sc-crystals overlapped with the melting of hc-crystals. Therefore, they attributed that the significant sc-crystallization signal obtained in the DSC thermograms was not originally present in the samples. However, there is an artifact of the crystallization of sc-crystals during heating, and these two opposite thermal effects were responsible for the small hc-crystal melting peaks. Since we could not reliably distinguish the sample’s crystallinity using DSC, WAXD was used to report the crystallinity of the samples as a function of degradation time.

**Figure 1:**
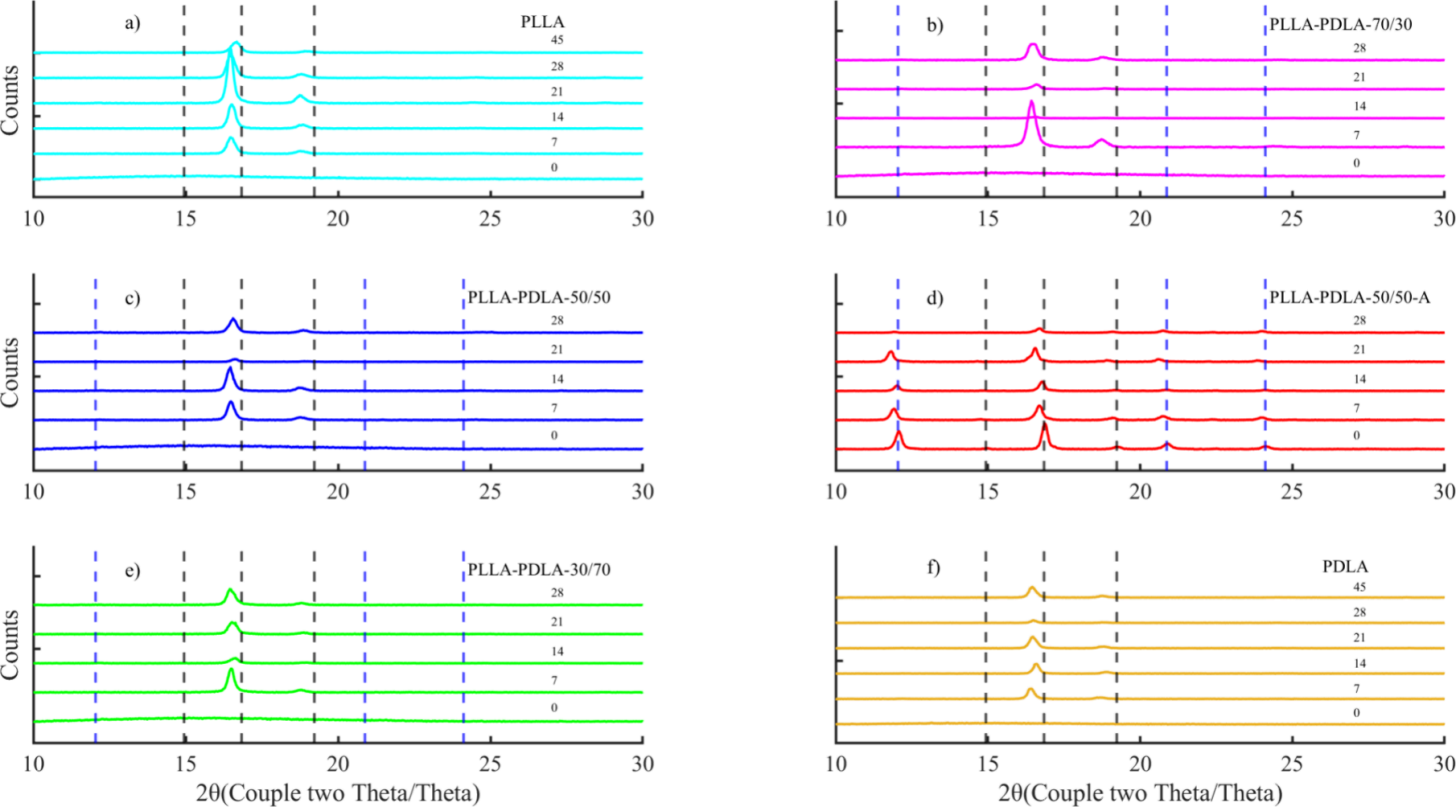
WAXD patterns of (a) PLLA; (b) PLLA/PDLA-70-30; (c) PLLA/PDLA-50–50; (d) PLLA/PDLA-50–50-A; (e) PLLA/PDLA-30–70; and (f) PDLA. In the WAXD patterns, the dashed black lines indicate the hc-crystals, and the dashed blue lines indicate the sc-crystals.

The WAXD patterns of the samples that could be collected during composting are shown in Figure 1. The background was removed from the patterns to facilitate visualization and remove the noise generated by the amorphous hallows. The hc-crystals mostly crystallize in peaks corresponding to α-crystals (2Θ at 14.9°, 16.8°, and 19.2° associated with the 010, 110/220, and 203 crystal planes) and sc-crystals (2Θ at 12.0°, 20.8°, and 24.1° associated with the 110, 300/030 and 200 crystal planes).^30^ Although some of the WAXD peaks are slightly shifted for the α-crystals, suggesting maybe an α’-crystal formation, the minor peaks at ∼20.0° and 24.0° on some of the thermograms seem to be unique to α-crystals. Additionally, no peaks less than 16.0° are shown. Hence, the most possible hc-crystallization seems to be α-crystals.^31^ The data depicted in Figure 1 are summarized in Table S3.

Figure 1a,f for PLLA and PDLA show the crystallization of hc-crystals, matching the information obtained by DSC for these samples in Figure S1a,f. The WAXD pattern of PDLA/PLLA-50–50-A shown in Figure 1d indicates the presence of hc-crystals and sc-crystals, as expected since it was initially annealed to induce the formation of stereocomplex and increased crystallization before biodegradation. Figure 1b,c,e for PLLA/PDLA-70–30, PLLA/PDLA-50–50, and PLLA/PDLA-30–70, respectively, show no presence of stereocomplex. There is no evidence of sc-crystal formation, confirming that the sc-crystals were crystallized during the DSC heating and overlapped with the melting point of the hc-crystals, as we discussed why the DSC analysis could not be used to report films’ crystallinity.^29^ However, Zhang et al. also reported the presence of double melting peaks, as evidenced by this process.^29^ In our work, we only observe the presence of double melting peaks on the PLLA/PDLA-50–50-A sample DSC thermogram (Figure S1d), which had already crystallized before exposure to compost. Hence, the initial presence of sc-crystals in PLLA/PDLA-50–50-A may catalyze additional sc-crystallization and a larger sc-crystal ratio. Future modulated DSC and WAXD studies will be needed to elucidate if additional sc-crystallization occurs during composting in the PLLA/PDLA-50–50-A sample.

### 3.2 Molecular Weight Evolution

**Figure 2:**
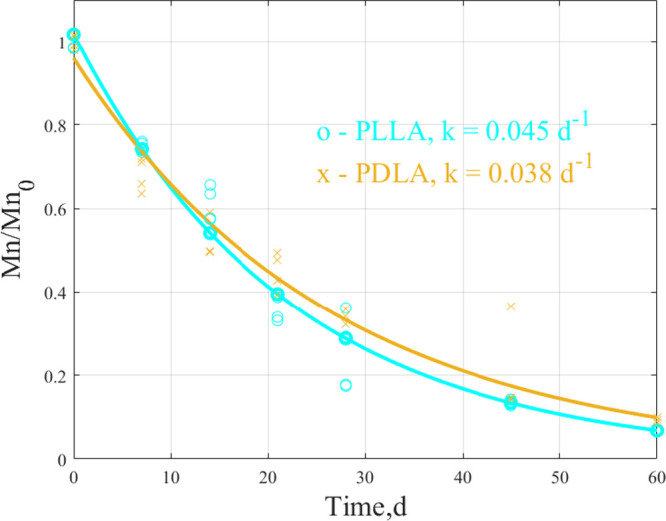
Normalized *M*_n_ reduction as a function of time for PLLA and PDLA was measured from biodegradation samples collected at each sampling time.

The normalized *M*_n_ reduction as a function of time for PLLA and PDLA in compost media is depicted in Figure 2. A Tukey–Kramer test showed no significant difference between the *k* values for PLLA and PDLA at *P* ≤ 0.05. The *M*_n_ for PLLA and PDLA dropped below 10 kDa around day 45, as presented in Table S4. Castro-Aguirre et al. demonstrated that examining changes in *M*_n_ may not be the best approach for looking at the evolution of PLA samples with different *M*_w_. However, examining changes in the MWD may be a better indicator of degradation behavior during the lag phase.^32^

**Figure 3:**
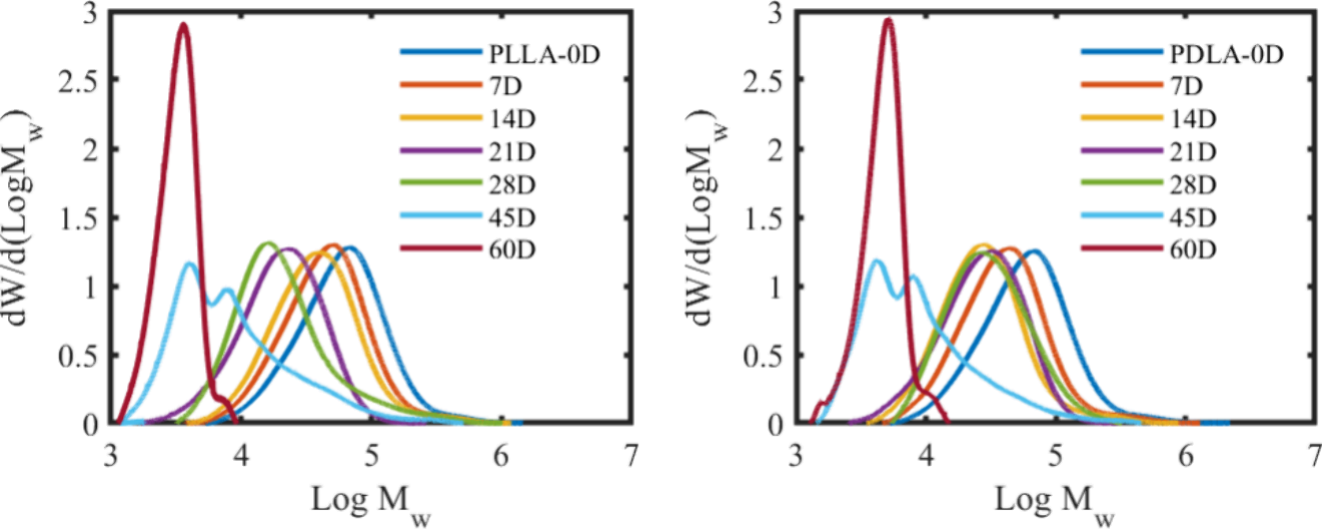
MWD of PLLA and PDLA at 58 ± 2 °C from days 0 to 60 was measured from biodegradation samples collected at each sampling time. This specific temperature was chosen to simulate the conditions of a composting environment, allowing us to observe the polymer’s behavior under these circumstances.

The MWD values as a function of time for PLLA and PDLA samples evaluated during biodegradation until day 60 are depicted in Figure 3. A shift in the MWD peak to the left signifies a decrease in the *M*_w_ due to hydrolysis, while the broadening of the peak is related to an increase in the polydispersity (*Đ*) due to chain scission.^33^ Both samples exhibited a multimodal distribution starting on day 45. At the beginning of hydrolysis, the *M*_W_ decreases because of ester bond breakdown, resulting in a shift in MWD to a lower *M*_w_. As time progresses, the MWD peak broadens because of an increased number of varying chain lengths within the polymer. By day 45, the bimodal distribution occurs due to differences in the chain lengths within the polymer as it degrades.^34,35^ The sharp peak d*W*/d(log *M*_w_) at log *M*_w_ 3.5 can be attributed to crystallization of the lower *M*_w_ PLLA and PDLA chains. Castro-Aguirre et al. and Limsukon et al. reported similar findings on the multimodal distribution and lower chain crystallization of PLA during hydrolytic degradation.^32,36^ After day 60, we could no longer collect film samples because they were too brittle and small. Table S4 summarizes the average *M*_w_ and *M*_n_ values at each sampling time.

### 3.3 Biodegradation in Simulated Composting Conditions

**Figure 4:**
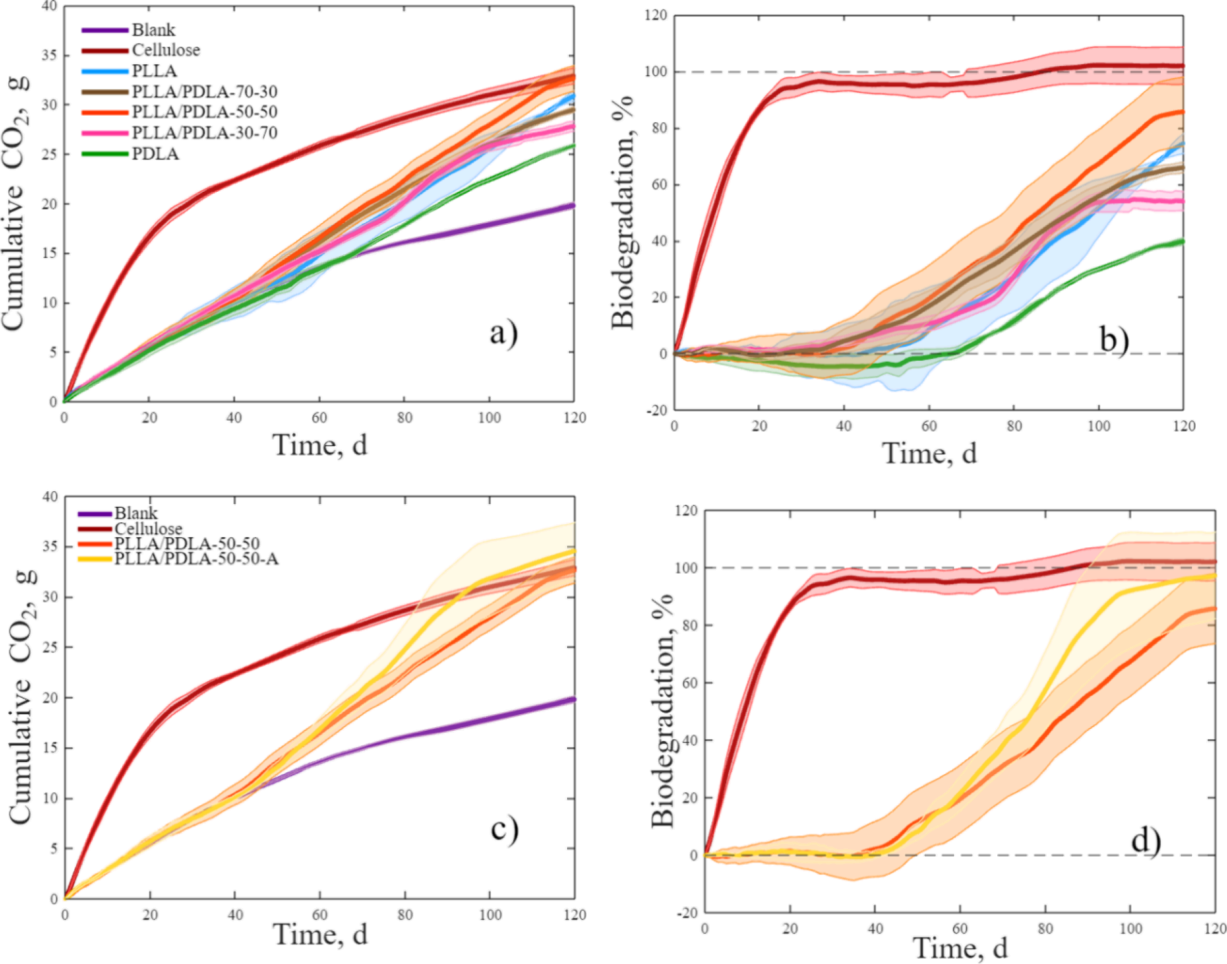
Simulated composting results of films in the bioreactors after 120 days of (a) CO_2_ evolution of blank, cellulose, PLLA, PLLA/PDLA-70–30, PLLA/PDLA-50–50, PLLA/PDLA-30–70, and PDLA; (b) percent biodegradation of cellulose, PLLA, PLLA/PDLA-70–30, PLLA/PDLA-50–50, PLLA/PDLA-30/70, and PDLA; (c) CO_2_ evolution of blank, cellulose, PLLA/PDLA-50/50, and PLLA/PDLA-50–50-A; and (d) percent biodegradation results of cellulose, PLLA/PDLA-50–50, and PLLA/PDLA-50–50-A.

CO_2_ evolution and biodegradation results during 120 days of testing are presented in Figure 4. Figure 4a,b shows the CO_2_ evolution and % biodegradation results, respectively, for the blank, cellulose, PLLA, PLLA/PDLA 70–30, 50–50, 30–70, and PDLA. Figure 4c,d shows the CO_2_ evolution and % biodegradation results, respectively, for the blank, cellulose, PLLA/PDLA 50–50, and 50–50-A. The % biodegradation data for each sample on days 0, 45, 60, 90, and 120 are summarized in Table S5.

Figure 4b,d shows the three phases of biodegradation—lag, biodegradation, and plateau. Cellulose reached almost 95% biodegradation by day 30. Cellulose’s hydrophilic nature and the activity of naturally occurring enzymes contribute to its breakdown so that it can be transferred through the cell wall of microorganisms to be easily assimilated by metabolic pathways.^17^ Cellulose powder has a very short lag phase with biodegradation starting almost immediately after the experiment begins.

In the case of PLLA and blends, all the film samples had an extended lag time of around 40–60 days, depending on the sample. There was no significant difference in the lag or hydrolysis phase of the amorphous films, as seen in Figure 4b, although the lag time of the PDLA samples was extended for a few days. The lag times shown exceeded the average 21 days previously reported.^17^ PLLA/PDLA 50–50-A, which initially contains sc-crystals, exhibits a more extended lag phase than does PLLA/PDLA 50–50.

After the lag phase, biodegradation starts, where microorganisms assimilate the broken-down oligomers, producing CO_2_ and water.^37^ Castro-Aguirre et al. determined that the *M*_n_ needed to drop below 10 kDa before assimilation could occur by the microorganisms after hydrolysis had been completed.^17^ This observation was evident in our samples: as the *M*_n_ dropped below that level for PLLA and PDLA around day 45, there was a notable transition from hydrolysis-dominant degradation to the biotic phase. Until day 45, no significant difference existed between the films in the lag phase. As the test progressed, the PDLA film showed a lower CO_2_ evolution than the other films. Research on the biodegradation of PDLA is limited, with only a few studies, such as one by Mbarki et al., focused on the biodegradation of PDLA/cellulose microfibers biocomposites.^22^ That study, conducted at mesophilic temperatures, did not include comparisons with PLLA or other materials. The authors concluded that crystallinity significantly influenced the biodegradation rate; the higher the crystallinity, the slower the biodegradation rate. As shown in Figure 4b, the biodegradation of the PLLA and PDLA films started increasing at different rates as the films crossed into the biotic biodegradation phase. The PLLA/PDLA-50–50 sample had the steepest increase, followed by PLLA/PDLA-70–30, PLLA, PLLA/PDLA-30–70, and PDLA. PDLA remained in the lag phase between 45 and 60 days. Since the PLLA/PDLA-30–70 sample is predominately PDLA, it is unsurprising that biodegradation evolves similarly to the PDLA sample. As the quantity of PDLA increased, the degradation rate slowed down.

The effect of annealing the PLLA/PDLA-50–50 films is seen in Figure 4d, which indicates that the crystallinity in the PLLA/PDLA50–50-A film slightly slowed down the hydrolytic degradation stage compared to the PLLA/PDLA-50–50 sample due to the initial presence of sc-crystals. Tsuji also reported that the hydrolysis resistance of PLA materials can be increased by stereocomplexation of PLLA and PDLA, with the rate being altered by the mixing ratio in solvent-cast films.^38^ Karst and Yang also reported that a PLLA/PDLA-50–50 blend had greater resistance to hydrolysis than PLLA or PDLA due to stronger hydrogen bonding and dipole–dipole interactions of the blend compared with pure PLLA or PDLA based on modeling scenarios from data collected by Tsuji.^39^ However, in our case, the PDLA films showed a higher resistance to degradation during both the abiotic and biotic stages. Loo et al. reported on the hydrolysis of annealed poly(lactide-*co*-glycolide), suggesting that the degree of crystallinity slows down hydrolytic degradation but only to a degree, after which the formation of voids due to annealing increases the rate of hydrolytic degradation.^40^ Pantani and Sorrentino reported on the influence of crystallinity on the biodegradation rate of injection-molded PLA samples in controlled composting conditions.^41^ They compared an amorphous sample to an injection-molded, annealed sample and found that crystallinity slowed the degradation rate initially and only partially affected it in the early stages of hydrolysis; however, crystallinity significantly affected the final swelling of the material and the subsequent biodegradation rate. It was concluded that the more compact structure of the annealed sample was less permeable to the enzymatic attack and breakdown of the oligomer diffusion; once the crystalline sample was broken down, the degradation rate became faster and similar to that of the amorphous sample.^41^ We observed similar results: degradation of the PLLA/PDLA-50–50-A sample started slowly but eventually was comparable to the degradation of the PLLA/PDLA-50–50 sample, as can be seen by the overlapping of the error bands, as indicated in Figure 4d. The annealing process aimed to provide initial crystallinity to the sample to improve the barrier properties for packaging applications.^42^ The evaluation of the biodegradation performance of this annealed sample, in comparison to blended PLLA/PDLA, does not show a detrimental effect on the overall biodegradation process, highlighting the potential of sc-PLA for packaging applications, which is known to be more heat resistant and has an potential enhanced water barrier than hc-PLA.

### 3.4 Surface Deterioration

**Figure 5:**
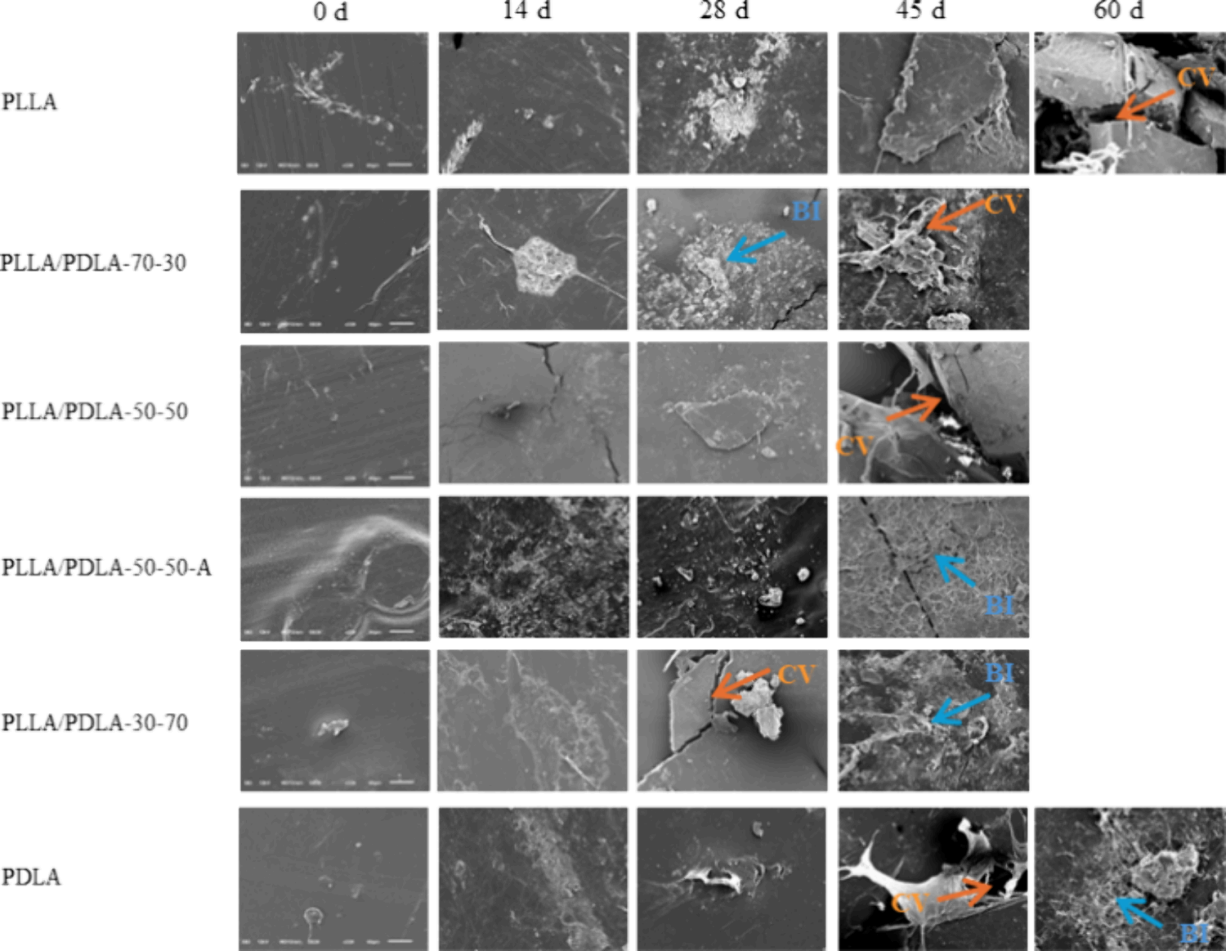
SEM micrographs of all the films at various experiment stages on days 0, 14, 28, 45, and 60 at 330× magnification. The bar on each image on day 0 represents 50 μm and applies to all of the micrographs. CV and arrow indicate the cavity, and BI and arrow indicate the biofilm.

SEM micrographs (330× magnification) of the films at each sample stage (days 0, 14, 21, 28, 45, and 60) are presented in Figure 5 and show how the films are progressively broken down as time evolves. There is minimal surface disturbance on day 0, except for the PLLA/PDLA-50–50-A sample, which had crystallization present at day 0. By day 14, tiny crystals had formed on the sample surfaces, especially on the PLLA/PDLA-50–50-A sample, which seems subject to preferential attack on the amorphous region. Between days 14 and 28, surface erosion can be observed in all samples. By days 45 and 60, depending on the sample, the surface was very rough and broken down due to the degradation that occurred, eroded mainly by hydrolysis at the early stages. Enzymatic degradation did not appear to begin until after intact film samples could no longer be collected. Figure S4 shows the SEM micrographs of each film at 5000 and 330× magnification in the same area of the sample. All micrographs at 5000× magnification show biofilm formation with cavities or holes on the film samples’ surface, indicating the hydrolysis effects. All films have a rough, uneven surface corresponding to what occurs at that later stage, biotic degradation of the increasing evolution of CO_2_, as indicated in Figure 4b,d. Mbarki et al. and Kijchavengkul et al. presented similar findings on SEM surface scans of PDLA and PBAT, respectively, where there was a progressive erosive change in the film surface from the beginning to the end of the experiment and progressive colonization of biofilms.^22,43^

Our research shows how PLLA, PDLA, several blends, and an annealed PLLA/PDLA-50–50 blended sample perform in a simulated biodegradation test. Most of the films were amorphous on day 0, except for the annealed one with an initial crystallinity of 16% (Table S3); however, by day 7, the blended films attained half of the crystallinity of the PLLA/PDLA-50–50-A film due to hc-crystallization. The PLLA/PDLA-50–50-A shows a minor increase on day 7, primarily due to amorphous depletion. Annealing appears to slow the initial abiotic hydrolysis stage; however, once the PLLA/PDLA-50–50-A film reaches the biodegradation phase, it recovers as the outer crystalline layers are broken down. The biodegradation of PDLA in a simulated compost environment has not previously been reported. PDLA degraded the slowest, reaching only 40% degradation by the end of the experiment at 120 days. Additionally, the presence of d-LA seems to make the blends more resistant to hydrolysis, although it is challenging to differentiate statistically due to the innate variation of biodegradation tests. These results provide a unique opportunity to tailor the degradation of PLLA/PDLA blends in pursuing PLA or any of its blended versions as more benign alternatives at the end of life.

## 4 Conclusions

This study explored the biodegradation of PLLA and PDLA films produced via cast extrusion, including multiple blends of PLLA/PDLA and an annealed PLLA/PDLA-50–50 sample. The results show that at the beginning of the experiment, in the lag phase, all of the films performed similarly until about day 45. The *X*_c_ of the PLLA, PDLA, and PLLA/PDLA films rose rapidly between days 0 and 7 but progressed slowly after the initial increase. In the PLLA/PDLA-50/50-A film, the hc-crystal portion seemed to break down faster than the sc-crystal portion, which may show that hc-crystals are more susceptible to hydrolysis than sc-crystals. We also saw a progressive decrease in the *M*_w_ as time progressed for both PLLA and PDLA, with a bimodal distribution occurring at day 45 due to differences in the chain lengths within the polymer as it degrades. The biodegradation results from the DMR show that all of the films were in the lag phase until between days 30 and 60. Then, the % biodegradation increased in all samples between days 30 and 120, with the annealed sample progressing the quickest, followed closely by the PLLA/PDLA-50–50 and PLLA samples. The biodegradation performance of the PLLA/PDLA blends varied primarily based on PDLA content, with pure PDLA achieving only about 40% degradation by the end of the testing period. The results indicate that as PDLA content increased, the degradation rate slowed down, with PDLA exhibiting the slowest and least biodegradation over 120 days. Given the limited research on PDLA, these findings provide new insights into the biodegradation behavior of various blended PLLA/PDLA films compared to pure PLLA. This study is particularly novel in its comprehensive biodegradation evaluation of cast-extruded PLLA, PDLA, and their film blends under identical conditions. It provides a deeper understanding of the biodegradation mechanisms, enhancing the potential for using PLA in commercial applications as a more sustainable alternative. Future research could explore the effects of annealing on different PLLA/PDLA blends to assess consistency in performance.

## Supporting Information Available

Additional raw material details and experimental results, including photographs and micrographs of the films at various experiment stages (PDF)
