# Supplementary material for: Composting Performance of l-Poly(lactic acid), d-Poly(lactic acid), Their Blends, and Stereocomplex PLA Films
Source: ACS Omega. 2025 May 1;10(18):18936–44. doi: 10.1021/acsomega.5c00985 (PMC12079277; doi:10.1021/acsomega.5c00985)
Supplement: Supplementary file 1 — ao5c00985_si_001.pdf [file ao5c00985_si_001.pdf]

Supplementary Information

Composting Performance of Stereocomplex Poly(lactic acid): Evaluating High Water Barrier Annealed Films for Biodegradation

James F. MACNAMARA Jr., Anibal BHER, Rafael AURAS[*]

School of Packaging, Michigan State University, East Lansing, MI 48824-1223, USA

*Corresponding author: aurasraf@msu.edu

Table S1. Average carbon content of samples measured by CHN elemental analyzer. 2
Table S2. Laboratory analysis of manure compost used for the biodegradation test.................... 2
Table S3. Crystallinity measured by XRD.. 3
Table S4. M_w and M_n for the films at times of sampling. 7
Table S5. Biodegradation (%) for each film at days 0, 45, 60, 90, and 120. 7

Figure S1. Photos of bioreactors at sampling days 7,14,21,28,45,60, and 90 for the various films. By day 60, the blended films were barely discernible; the same holds for PLLA and PLDA by day 90.. 4
Figure S2. DSC thermograms of all tested films at day 0 from 50 to 250 °C. 5
Figure S3: DSC thermograms of various films from 75 to 250 °C measured from biodegradation samples collected at each sampling time: **a)** PLLA; **b)** PLLA/PDLA-70-30; **c)** PLLA/PDLA-50-50; **d)** PLLA/PDLA-50-50-A; **e)** PLLA/PDLA-30-70; **f)** PDLA. The dashed black lines represent the HC-crystals’ melting point, and the dashed blue lines represent the SC-crystals’ melting point. 6
Figure S4. SEM micrographs of all the films on the last sampling day (either day 45 or day 60). For each film type, the micrograph on the left is at 5000× magnification, and the one on the right is at 330× magnification. The bar in each micrograph signifies the scale: the bar at 5000× represents 5 microns, and the bar at 330× represents 50 microns. The arrows represent the area that is being magnified between the two micrographs.. 8

Table S1. Average carbon content of samples measured by CHN elemental analyzer.

Sample	Average Carbon content (%)		Standard Deviation (%)
Cellulose	42.50	±	0.08
PLLA	50.59	±	0.14
PLLA/PDLA-70-30	50.43	±	0.13
PLLA/PDLA-50-50	50.88	±	0.24
PLLA/PDLA-50-50-A	51.51	±	0.38
PLLA/PDLA-30-70	49.87	±	0.84
PDLA	51.56	±	0.22

Table S2. Laboratory analysis of manure compost used for the biodegradation test.

Compost				
**Tests**	**Units**	**Desired Range**	**Results**	**Interpretation**<br>**Low Desired High**
**pH**		6.0 – 8.0	7.22	**X**
**E. C. - Saturation Paste**	mmho/cm	< 4	13.8	**X**
**Nitrate-N (NO_3-N)***	ppm	40 – 99		
**Ammonium-N (NH_4-N)***	ppm			
**Total Dry Solid**	%		55.7	**X**
**Total Volatile Solid**	%		44.4	
**C/N Ratio**		<25	10.3	**X**
**Tests**	**Units**		**Wet Weight Basis**	
**Total Nitrogen (N)**	%		2.27	
**Total Phosphorus (P)**	%		1.36	
**Total Potassium (K)**	%		1.46	
**Total Calcium (Ca)**	%		6.11	
**Total Magnesium (Mg)**	%		1.57	
**Total Zinc (Zn)**	ppm		380	
**Total Iron (Fe)**	ppm		7033	
**Total Manganese (Mn)**	ppm		294	
**Total Copper (Cu)**	ppm		126	
**Total Carbon (C)**	%		23.3	
**Total Sodium (Na)**	%		0.347	
**Total Aluminum (Al)**	%		0.198	
**Total Sulfur (S)**	%		0.505	
**Total Boron (B)**	ppm		39	

*Interpretation for nitrate-N is for growing media only. If this material is to be used as soil amendment, the interpretation for nitrate-N is not applicable.

Table S3. Crystallinity measured by XRD in %.

Time	L130	70-30	50-50	50-50A	30-70	D120
D0	0	0	0	16	0	0
D7	10	6	9	20	6	8
D14	13	6	10	16	2	11
D21	5	7	8	20	9	13
D28	16	9	8	15	11	11

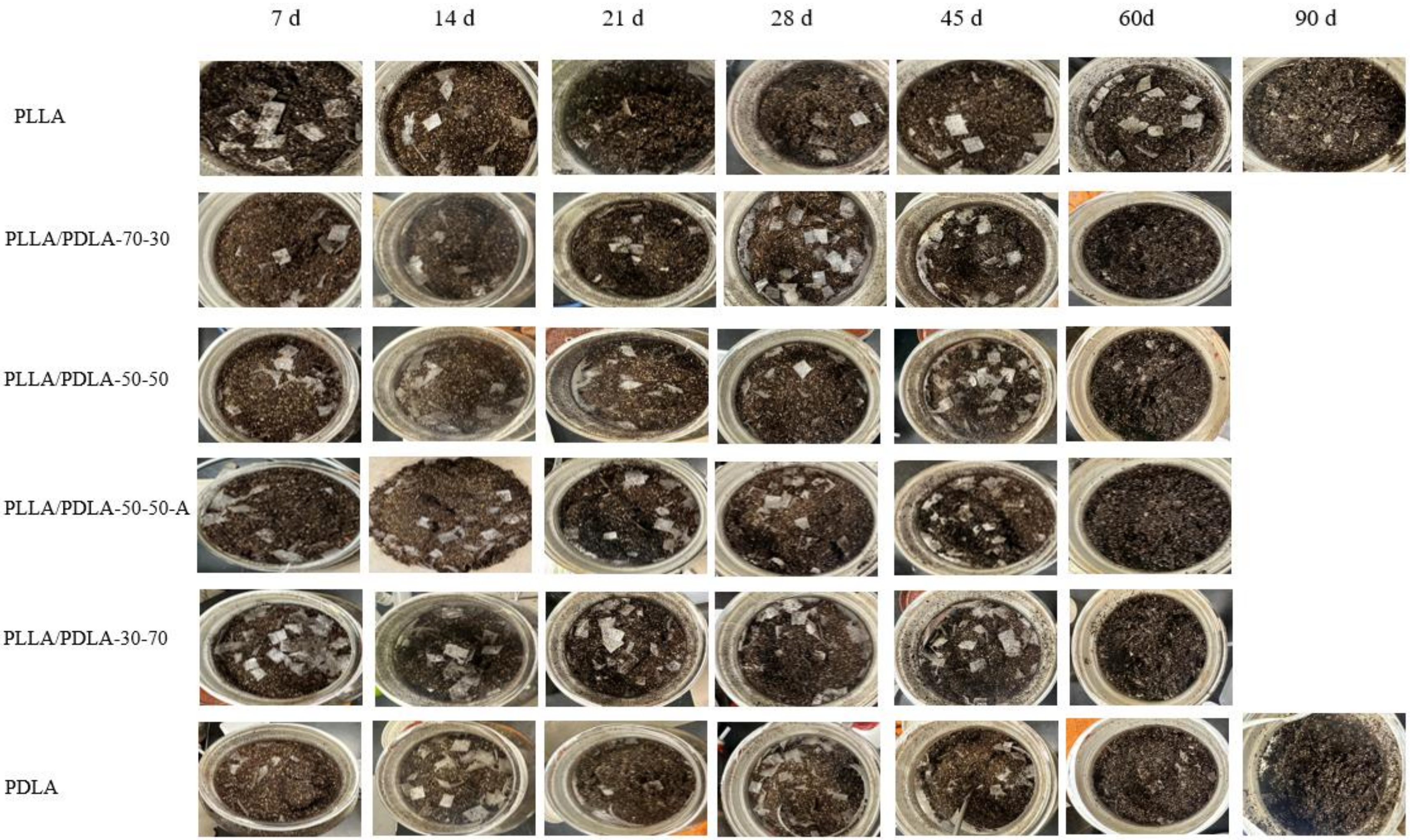

Figure S1. Photos of bioreactors at sampling days 7,14,21,28,45,60, and 90 for the various films. By day 60, the blended films were barely discernible; the same holds for PLLA and PLDA by day 90.

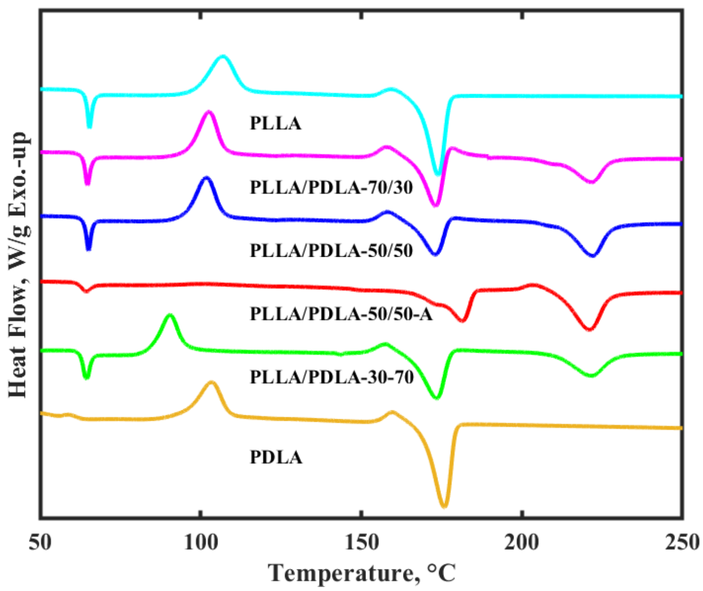

Figure S2. DSC thermograms of all tested films at day 0 from 50 to 250 °C.

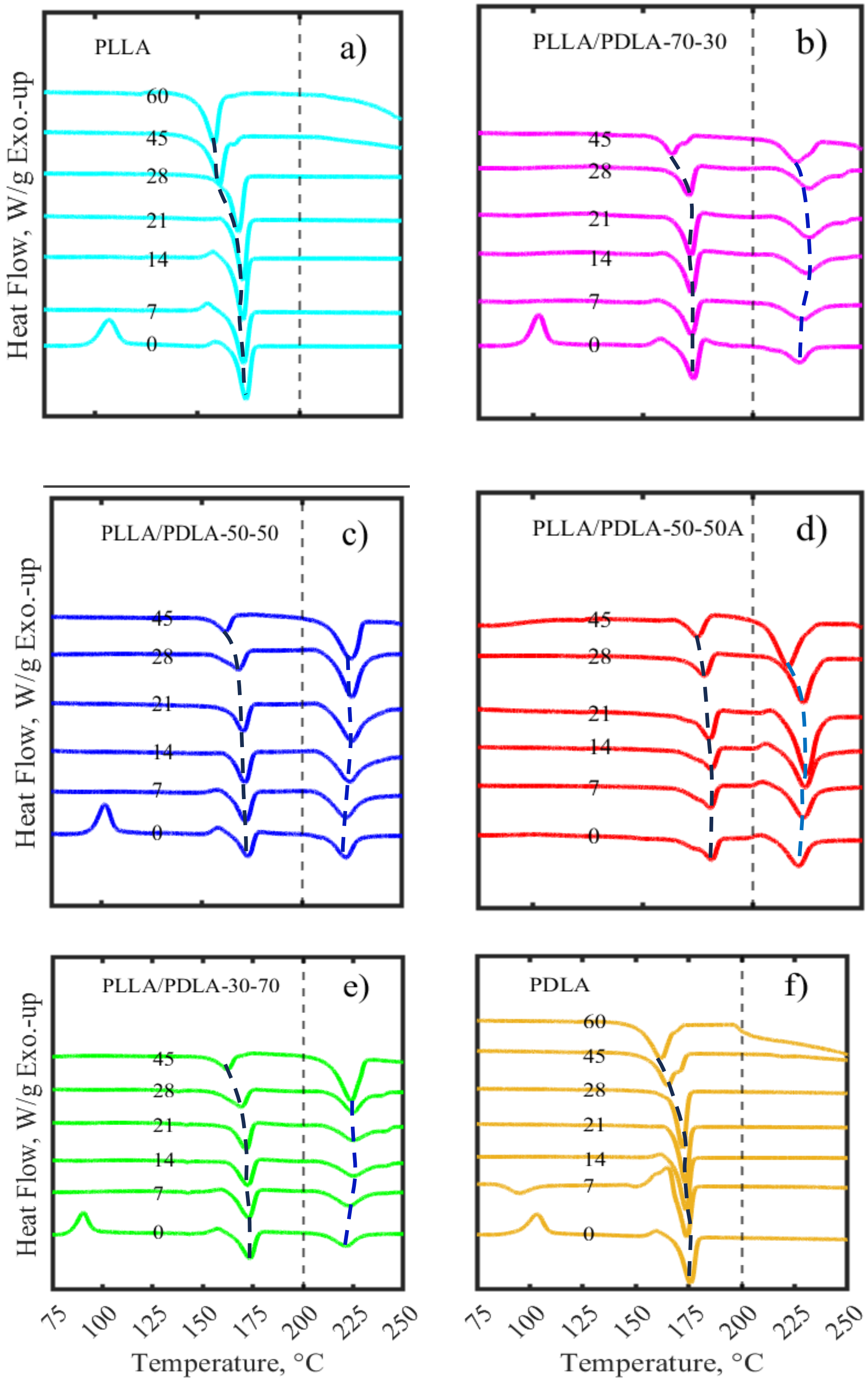

Figure S3: DSC thermograms of various films from 75 to 250 °C measured from biodegradation samples collected at each sampling time: **a)** PLLA; **b)** PLLA/PDLA-70-30; **c)** PLLA/PDLA-50-50; **d)** PLLA/PDLA-50-50-A; **e)** PLLA/PDLA-30-70; **f)** PDLA. The dashed black lines represent the HC-crystals' melting point, and the dashed blue lines represent the SC-crystals' melting point.

Table S4. M_w and M_n for the films at times of sampling.

	M_n (kDa)				M_w (kDa)			
	**PLLA**		**PDLA**		**PLLA**		**PDLA**	
Day	Average	St Dev.	Average	St Dev.	Average	St Dev.	Average	St Dev.
0	44951	798	42882	787	82641	2313	79999	2313
7	33535	593	29218	1739	60952	2393	54113	5630
14	27458	1858	23618	2008	51098	4537	43494	6245
21	17542	1437	19217	1956	31219	3360	31936	3244
28	11489	3161	14841	752	25932	1422	25372	1954
45	6084	315	7492	1636	16300	1837	20777	8038
60	3002	12	4011	256	3491	29	4782	157

Table S5. Biodegradation (%) for each film at days 0, 45, 60, 90, and 120.

		% Biodegradation											
**Sample**	**0 d**	**45 d**			**60 d**			**90 d**			**120 d**		
Cellulose	0	95	±	3	95	±	4	101	±	6	102	±	7
PLLA	0	0	±	2	9	±	15	41	±	14	75	±	4
PLLA/PDLA-70-30	0	7	±	5	17	±	5	47	±	5	66	±	2
PLLA/PDLA-50-50	0	6	±	11	20	±	12	56	±	12	86	±	12
PLLA/PDLA-50-50-A	0	3	±	2	21	±	6	80	±	20	97	±	15
PLLA/PDLA-30-70	0	6	±	2	11	±	3	45	±	3	54	±	3
PDLA	0	-5	±	4	-1	±	3	22	±	1	40	±	1

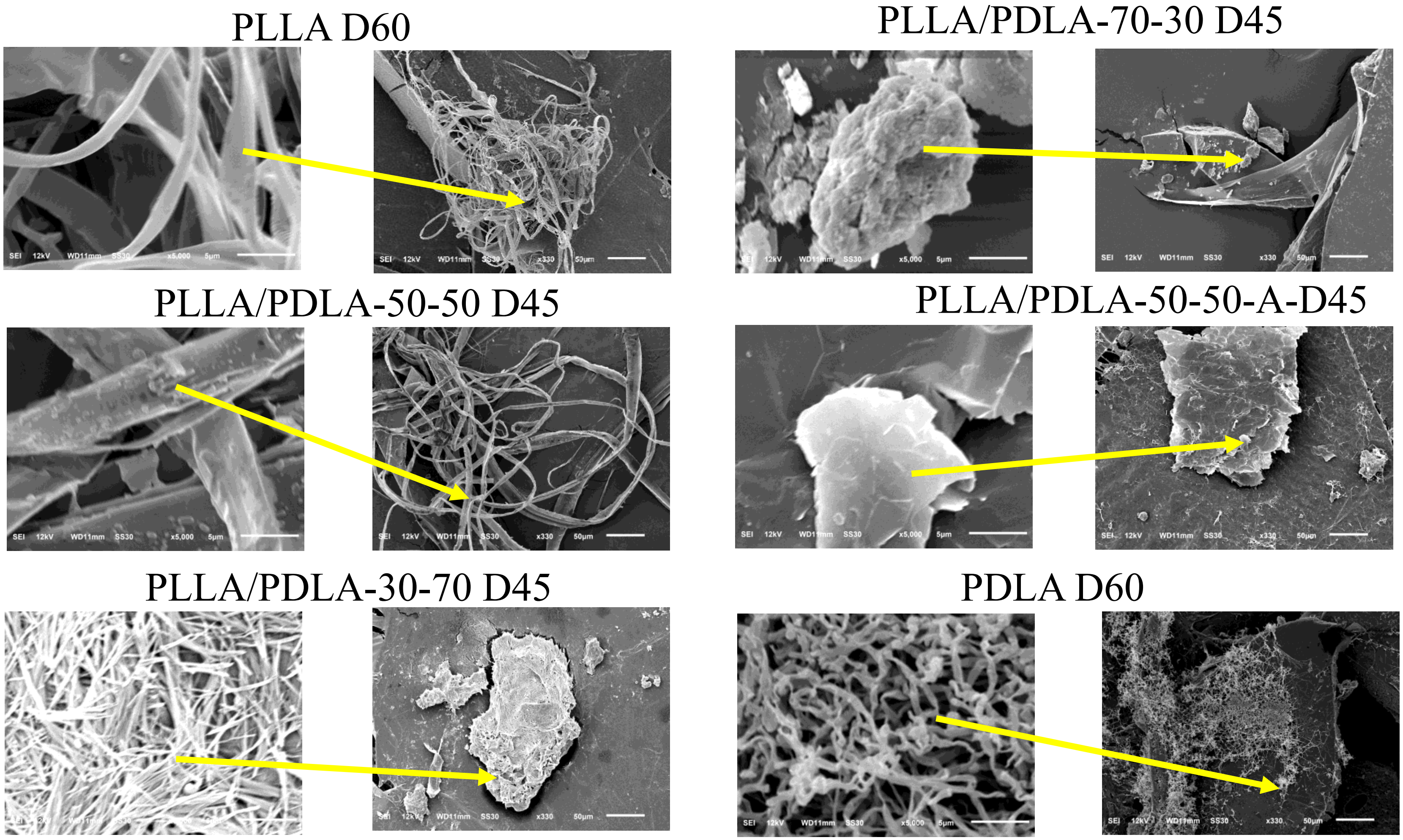

Figure S4. SEM micrographs of all the films on the last sampling day (either day 45 or day 60). For each film type, the micrograph on the left is at 5000× magnification, and the one on the right is at 330× magnification. The bar in each micrograph signifies the scale: the bar at 5000× represents 5 microns, and the bar at 330× represents 50 microns. The arrows represent the area that is being magnified between the two micrographs.
